# SARS-CoV-2 IgG antibody responses in rt-PCR-positive cases: first report from India

**DOI:** 10.1099/acmi.0.000267

**Published:** 2021-10-21

**Authors:** Girish Chandra Dash, Debaprasad Parai, Hari Ram Choudhary, Annalisha Peter, Usha Kiran Rout, Rashmi Ranjan Nanda, Jaya Singh Kshatri, Srikanta Kanungo, Subrata Kumar Palo, Nityananda Mandal, Sanghamitra Pati, Debdutta Bhattacharya

**Affiliations:** ^1^​ Department of Microbiology, ICMR-Regional Medical Research Centre (Dept of Health Research, Ministry of Health and Family Welfare, Govt. of India), Chandrasekharpur, Bhubaneswar-751023, India

**Keywords:** antibody, COVID-19, *C*_t_ value, IgG, RT-PCR, SARS-CoV-2

## Abstract

**Introduction:**

Severe acute respiratory syndrome coronavirus 2 (SARS-CoV-2) antibody responses remain poorly understood and the clinical utility of serological testing is still unclear.

**Aim:**

To understand the relationship between the antibody response to SARS-CoV-2 infection and the demographics and cycle threshold (*C*_t_) values of confirmed RT-PCR patients.

**Methodology:**

A total of 384 serum samples were collected from individuals between 4–6 weeks after confirmed SARS-CoV-2 infection and tested for the development of immunoglobulin class G (IgG) against SARS-CoV-2. The *C*_t_ values, age, gender and symptoms of the patients were correlated with the development of antibodies.

**Results:**

IgG positivity was found to be 80.2 % (95 % CI, 76.2–84.2). Positivity increased with a decrease in the *C*_t_ value, with the highest (87.6 %) positivity observed in individuals with *C*_t_ values <20. The mean (±sd) *C*_t_ values for IgG positives and negatives were 23.34 (±6.09) and 26.72 (±7.031), respectively. No significant difference was found for demographic characteristics such as age and sex and symptoms and antibody response. The current study is the first of its kind wherein we have assessed the correlation of the RT-PCR *C*_t_ with the development of IgG against SARS-CoV-2.

**Conclusion:**

Although *C*_t_ values might not have any relation with the development of symptoms, they are associated with the antibody response among SARS-CoV-2-infected individuals.

## Introduction

An outbreak of pneumonia was reported in Wuhan, Hubei Province, PR China in late December 2019 [1], and was later identified to be caused by a novel beta coronavirus closely related to the severe acute respiratory syndrome (SARS) coronavirus (CoV) family – severe acute respiratory syndrome coronavirus 2 (SARS-CoV-2) [2]. As of 30 October 2020, more than 51.8 million individuals were infected with SARS-CoV-2, with 1.28 million SARS-CoV-2-associated deaths [3]. The USA, India and Brazil account for the majority of the cases worldwide, with India accounting for 8.2 million cases and 1.2 million deaths [4].

There is a scarcity of information on the antibody response to SARS-CoV-2 infection [5]. SARS-CoV-2 antibodies have been detected from a range of a few days to 3 weeks after onset of symptoms, with the median time reported as 6 days for detectable levels of immunoglobulin class G (IgG) [6–8]. The presence of SARS-CoV-2 IgG antibodies, which is indicative of current or previous infection by SARS-CoV-2, is thought to confer some degree of immunity [9], although there is uncertainty regarding the duration and extent of immunity conferred by them [8, 10, 11].

The present study carried out semi-quantitative SARS-CoV-2 IgG antibody estimation to understand the body’s antibody response in correlation with the severity of SARS-CoV-2 symptoms, cycle threshold (*C*_t_) value, gender and age.

## Methodology

### Sample collection

A subset of 384 individuals were included in the study to evaluate SARS-CoV-2 IgG between 4 and 6 weeks after being confirmed positive for SARS-CoV-2 by real-time reverse transcription-polymerase chain reaction (RT-PCR) from the month of August to October 2020. The *C*_t_ values, age, gender and symptoms of the patients were correlated with the development of antibodies. Confirmed coronavirus disease 2019 (COVID-19) cases were defined as those that tested positive for SARS-CoV-2 RNA using RT-PCR testing of combined nasopharyngeal and throat swab (NT) samples. Patients who presented with one or more symptoms, such as fever, breathlessness, cough, fatigue, muscle pain, clogged nasal cavity, sore throat, diarrhoea, loss of taste (anosmia) and loss of smell (ageusia), during the time of RT-PCR testing were considered symptomatic. This study was approved by the Ethics Committee of ICMR-Regional Medical Research Centre, Bhubaneswar. We obtained informed consent from all participants.

### Testing for SARS-CoV-2 IgG

Semi-quantitative SARS-CoV-2 IgG testing was performed using the ARCHITECT i2000SR platform, which uses chemiluminescent microparticle immunoassay (CMIA) technology for the detection of IgG antibodies against the nucleocapsid protein of SARS-CoV-2 from human serum. The cutoff for antibody response was 1.4 index, above which the sample was considered positive.

### Data analysis

Data were entered using MS Excel and descriptive statistical analysis was performed using SPSS software (IBM SPSS for Windows, version 24.0, Armonk, NY, USA). Scatterplots were used to demostrate the relationship between the antibody titre and *C*_t_ values. A linear treadline was used to show the corelation. Qualitative data were described using frequencies and percentages and analysed using the chi-square test. Quantitative data were described using mean and standard deviation (sd) and analysed using an independent sample *t*-test. A *P*-value of less than 0.05 was considered to be statistically significant.

## Results

**Fig. 1.**
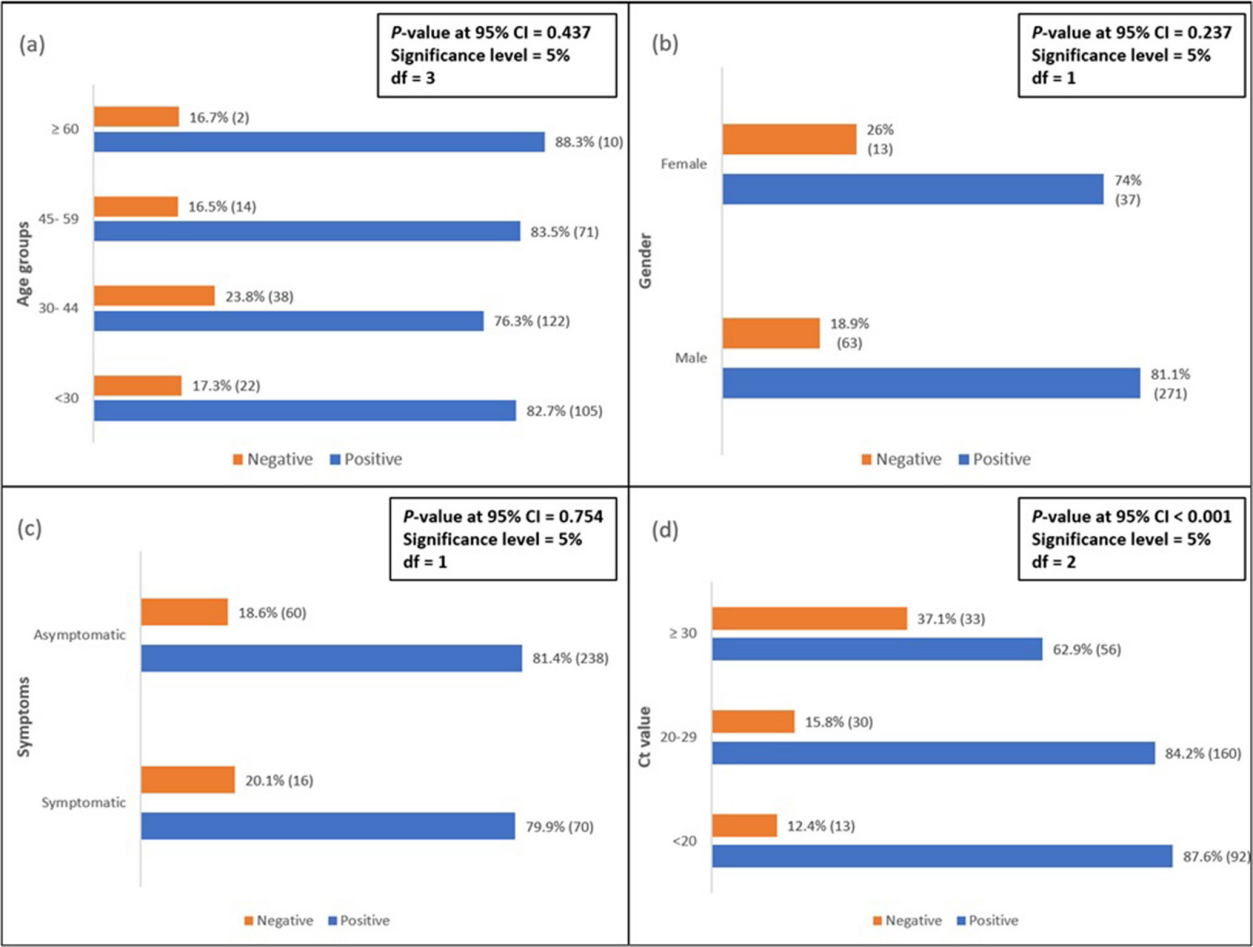
Association between demographic characteristics and *C*_t_ value with IgG antibody response. (**a**) Percentages of IgG results in various age groups. (**b**) Percentages of IgG results in males and females. (**c**) Percentages of IgG results in symptomatic and asymptomatic cases. (**d**) Percentages of IgG results in various *C*_t_ value groups, CI, confidence interval; df, degree of freedom.

**Fig. 2.**
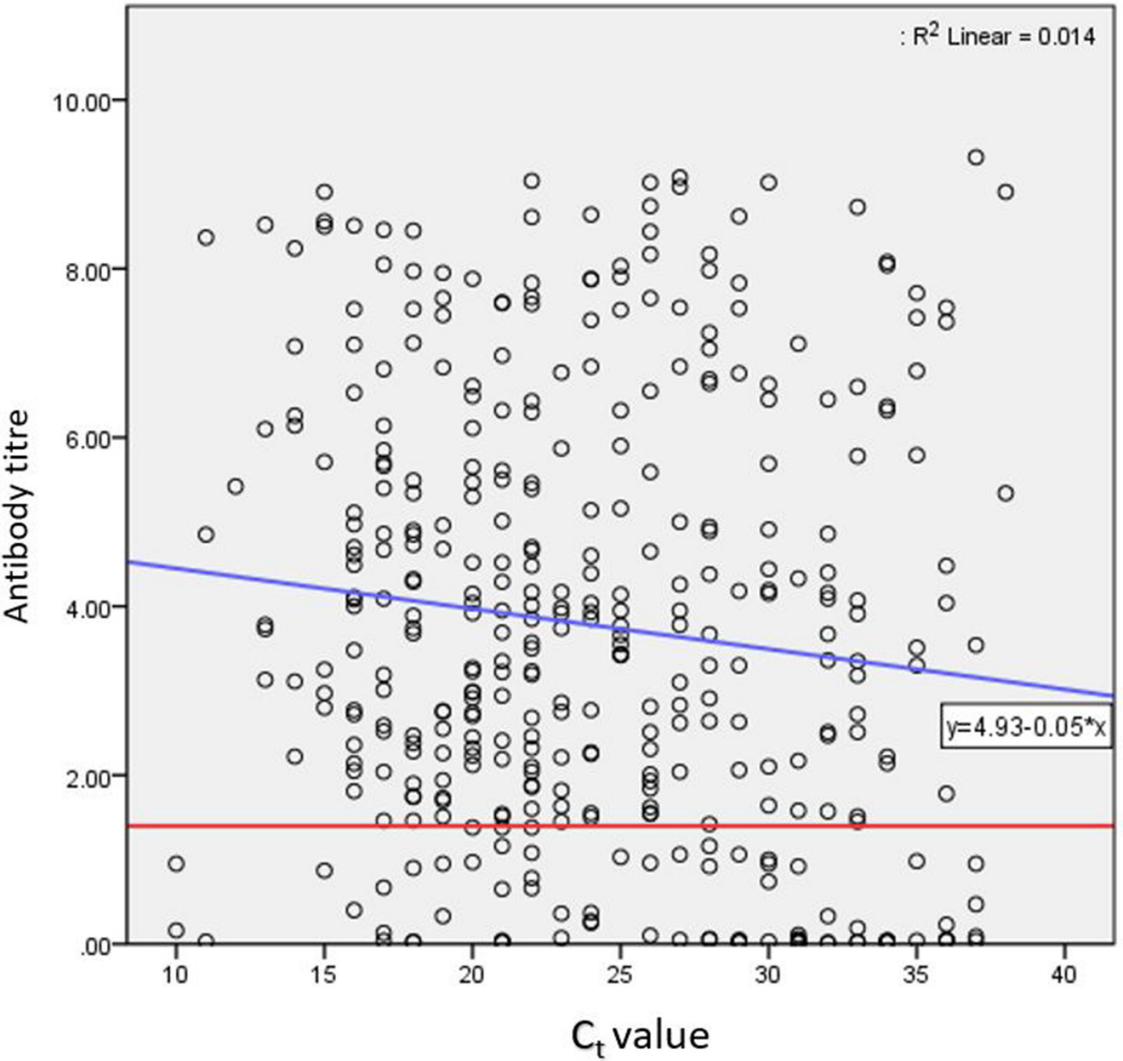
Antibody titre vs *C*_t_ value for all 384 COVID-19 patients. Red line indicates the reactive titre value of 1.4 index.

Out of the total 384 samples collected from SARS-CoV-2 rt-PCR-positive individuals, 80.2 % (95 % CI, 76.2–84.2) of the samples were found to be positive for antibodies against SARS-CoV-2. The median time of the sample collection was 34 days after confirmatory RT-PCR testing. The mean age of the IgG-positive and -negative individuals was 36.94±11.29 and 36.09±10.18, respectively. IgG positivity was found to be highest (88.3 %) in persons aged ≥60 years. No significant difference for antibody response was detected in different age groups (*P*=0.437) (Fig. 1a). The samples were collected predominantly from males [*n*=334 (86.9 %)] as opposed to females [*n*=50 (13.1 %)]. Males had a greater chance of producing antibodies than females after a SARS-CoV-2 infection, but the difference was found to be statistically insignificant (*P*=0.237) (Fig. 1b). There was no difference in the mean antibody titre values between male and female groups (*P*=0.836). The mean *C*_t_ values of symptomatic and symptomatic patients were 23.48±6.070 and 24.16±6.521, respectively. There was no statistical difference for IgG response between symptomatic and asymptomatic patients (*P*=0.754) (Fig. 1c). The mean (±sd) *C*_t_ value of the IgG Ab positives was 23.34 (±6.09) and in IgG negatives it was 26.72 (±7.031). The difference in the mean values was found to be statistically significant (*P*<0.001). The percentage of IgG Ab positives increased with a decrease in the *C*_t_ value, which was found to be statistically significant (*P*<0.001) (Fig. 1d). The antibody titre values of the positive individuals mostly (71 %) presented between 1.4 to 6.0 index (Fig. 2).

## Discussion

In this second phase of the COVID-19 pandemic, sero-testing has emerged as a very useful platform to track down the susceptible population. This method is fast and is considered to be complementary to the gold standard RT-PCR test. Most studies have found a surprisingly lower IgG prevalence (≤90 %) among recovered patients, although a small part of the literature has suggested a higher percentage for the same [8, 9]. This anomaly requires a study based on patients’ demographics, infection severity and viral load.

In this study, the antibody response was found to be 80.2 % among COVID-19-positive individuals, which had been reported by most of the literature [8, 9]. However, a statistically significant correlation was found between *C*_t_ value and IgG antibody titre. Antibody titre was found to be directly proportional to a lower *C*_t_ value (indicative of higher viral load). Hence, it can be said that higher viral load might lead to the development of a stronger immune response in a SARS-CoV-2-infected individual. Although the kind of immunity exerted by IgG is not yet properly understood, some level of immunity is definitely conferred by IgG, as found in this study. Similar to earlier studies [8], our study showed that the IgG response was greater in males than in females, but the difference was statistically insignificant. The predominantly male population could be a limitation of the current study in determining IgG prevalence in different sexes. There was also no statistically significant association between *C*_t_ value and the development of symptoms. One of the earlier studies found that antibody titre cannot be correlated with SARS-CoV-2 disease severity, which can also be corroborated by these data [12].

Without the use of a standard curve using reference materials, the *C*_t_ value by itself cannot be interpreted directly as viral load [13], but *C*_t_ can be used as being indicative of viral load in an infected individual.

There are additional implications from our study for blood banks wherein donors are screened for antibodies using qualitative antibody tests for convalescent plasma to treat COVID-19 patients. To support a previous diagnosis of SARS-CoV-2, these facilities have often relied on self-reporting about patient history and onset of symptoms. The correlation of *C*_t_ values with a semi-quantitative SARS-CoV-2 IgG assay can provide significant assistance in plasma donor selection.

## Conclusion

The current study is the first of its kind wherein we have assessed the correlation of RT-PCR *C*_t_ with the development of IgG against SARS-CoV-2. The *C*_t_ value might not have any relation with the severity of the disease, but is associated with the antibody response in SARS-CoV-2-infected persons. However, further long-term studies of longitudinal follow-up of a cohort will help in improving our understanding and forming definitive conclusions.
